# Current peptide vaccine and immunotherapy approaches against Alzheimer's disease

**DOI:** 10.1002/pep2.24289

**Published:** 2022-06-24

**Authors:** Chelsea Marie T. Parrocha, James S. Nowick

**Affiliations:** ^1^ Department of Pharmaceutical Sciences University of California Irvine Irvine California USA; ^2^ Department of Chemistry University of California Irvine Irvine California USA

**Keywords:** β‐amyloid, Alzheimer's disease, immunotherapy, peptide vaccine, tau

## Abstract

Peptide vaccines and immunotherapies against aggregating proteins involved in the pathogenesis and progression of Alzheimer's disease (AD)—the β‐amyloid peptide (Aβ) and tau—are promising therapeutic avenues against AD. Two decades of effort has led to the controversial United States Food and Drug Administration (FDA) approval of the monoclonal antibody Aducanumab (Aduhelm), which has subsequentially sparked the revival and expedited review of promising monoclonal antibody immunotherapies that target Aβ. In this review, we explore the development of Aβ and tau peptide vaccines and immunotherapies with monoclonal antibodies in clinical trials against AD.

## Graphical abstract

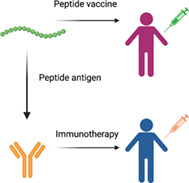

## 1 INTRODUCTION

Alzheimer's disease (AD) is the most common form of dementia, affecting ca. 6.2 million Americans.^[^ ^1^ ^]^ It is expected to increase to 13.8 million by 2060. Four cognition‐enhancing drugs have been used to treat the symptoms of AD, by inhibiting cholinesterase (rivastigmine, galantamine, and donepezil) or targeting the NMDA receptor (memantine).^[^ ^2^ ^]^ Psychotropic agents have also been used to treat behavioral disturbances. These small‐molecule drugs do not prevent the inevitable decline associated with AD but only mitigate the symptoms.^[^ ^2^ ^]^ There are currently no therapeutic agents that substantially alter the progression of the disease in the growing number of patients diagnosed with AD.

Peptide vaccines and immunotherapies with monoclonal antibodies have thus far demonstrated promise in slowing the cognitive decline associated with AD. Vaccines involve administration of an antigen associated with the disease. The annual flu shot or the Johnson & Johnson vaccine against COVID‐19 are typical examples. In vaccination, a patient's immune system is trained to produce antibodies against a pathogen or molecule associated with the disease. Immunotherapies involve administration of exogenously produced antibodies that recognize molecules or pathogens associated with the disease. HUMIRA is a monoclonal antibody that neutralizes tumor necrosis factor α to reduce inflammation associated with rheumatoid arthritis and other inflammatory disease. Immunotherapy does not involve training the immune system, but instead just provides the antibodies needed to treat the disease.

The vaccines and immunotherapies that have been pursued thus far for AD have focused on sequestering and clearing the aggregating peptides and proteins involved in the pathogenesis and progression of the disease, the β‐amyloid peptide (Aβ) and tau. In AD, Aβ aggregates to form fibrils and plaques in the brain,^[^ ^3^ ^]^ and hyperphosphorylated tau aggregates to form neurofibrillary tangles.^[^ ^4^ ^]^ Aβ, tau, and peptide fragments thereof have been used in peptide vaccine and immunotherapy development against AD. This review will cover the design and development of peptide vaccines and immunotherapies with a peptide or protein that contributes to the progression and pathogenesis of AD.

## 2 β‐AMYLOID PEPTIDE VACCINES

The earliest Aβ peptide vaccine, AN‐1792, consisted of the Aβ_1–42_ peptide formulated with the QS21 adjuvant.^[^ ^5^ ^]^ The AN‐1792 vaccine was designed to train the immune system to generate antibodies that target and sequester endogenous Aβ and thus prevent the formation of Aβ plaques in the brain and the associated cognitive decline.

Even though the AN‐1792 vaccine showed promising safety and tolerability in a phase I clinical trial which began in 2001, the vaccine failed 2 years later in a phase II clinical trial because it induced meningoencephalitis, a form of brain inflammation.^[^ ^6^ ^]^ Subsequent studies have suggested that the QS21 adjuvant may have exacerbated the inflammatory T‐cell response—a Th‐1 response—that produced the meningoencephalitis.^[^ ^3^ ^]^ Epitope mapping and additional studies of Aβ_1–42_ further established that the central region, between residues 14 and 34, induces a Th‐1 response.^[^ ^6^, ^7^ ^]^ By contrast, the *N*‐terminal region of Aβ, between residues 1 and 15, induces the production of antibodies against Aβ through a B‐cell response—a Th‐2 response.^[^ ^6^, ^7^ ^]^ A follow‐up study, performed 14 years after the phase I clinical trial, showed plaque removal associated with the antibodies that AN‐1792 induced.^[^ ^7^, ^8^ ^]^ Although the AN‐1792 peptide vaccine failed in clinical trials, it inspired the second generation of Aβ peptide vaccines.

**FIGURE 1:**
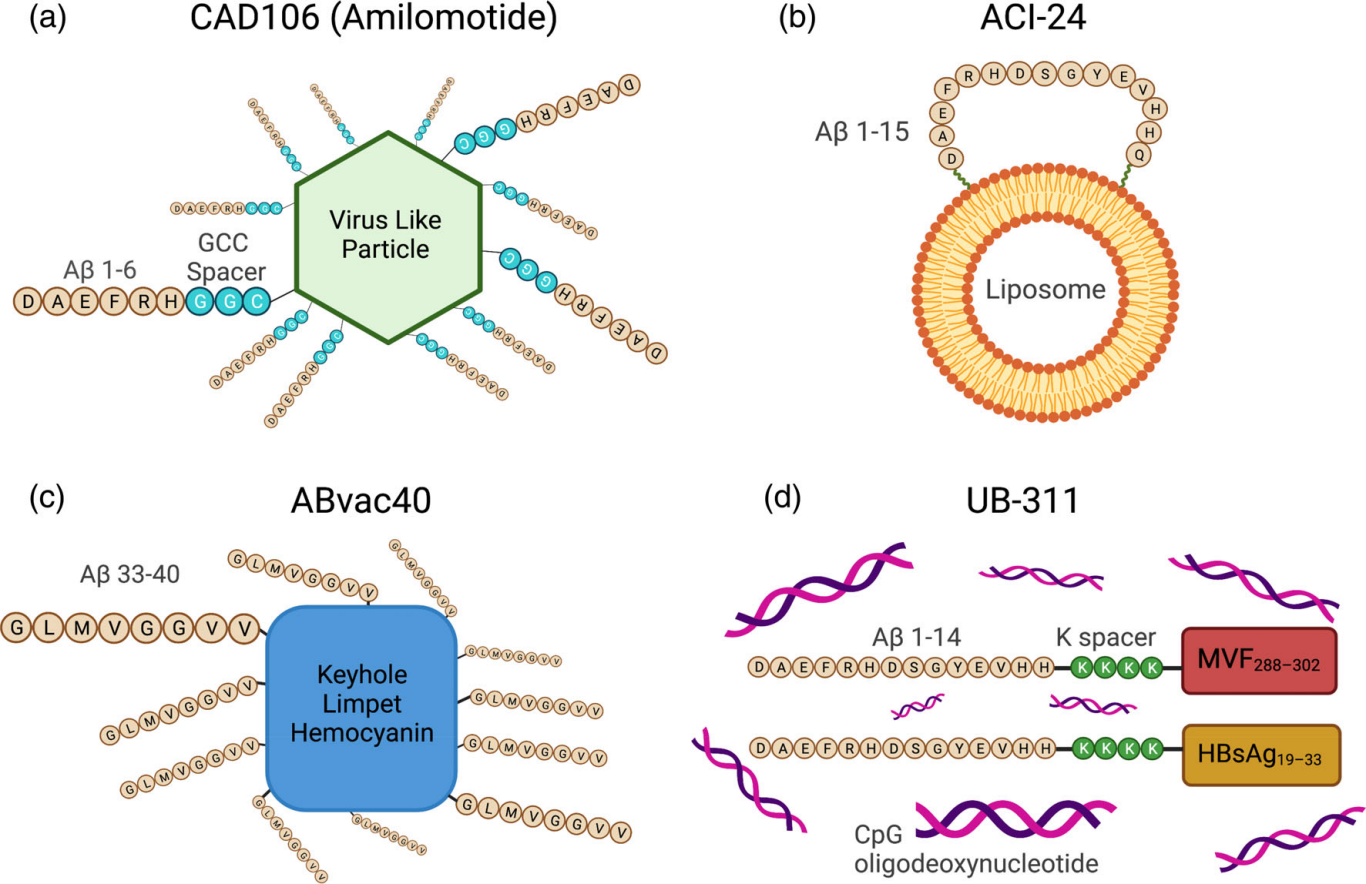
Four Aβ peptide vaccines that have advanced to phase II clinical trials. (a) CAD106, (b) ACI‐24, (c) ABvac40, and (d) UB‐311

Nine Aβ peptide vaccines have subsequently entered clinical trials (Table S1). Figure 1 summarizes the four most promising peptide vaccine candidates tested in at least phase II clinical trials (CAD‐106, ACI‐24, ABvac40, and UB‐311) and illustrates the region of Aβ that was used as an antigen and the means of antigen presentation. Most of the second‐generation peptide vaccines were designed with the *N*‐terminus of Aβ to direct a Th‐2 response while not inducing a Th‐1 response, thus building on the lessons learned from the failure of the AN‐1792 peptide vaccine.

CAD‐106 (Amilomotide: Novartis Pharmaceuticals) is the only AD peptide vaccine to enter phase II/III clinical trials. CAD‐106 contains Aβ_1–6_ followed by a three amino acid spacer (GGC) conjugated to an *Escherichia coli* RNA phage Qβ virus‐like particle (VLP) delivery system.^[^ ^9^ ^]^ The VLP promotes multivalent antigen presentation of Aβ peptide fragments (Figure 1a). The peptide vaccine was well tolerated and generated an immune response. In a phase IIB clinical trial, PET imaging studies showed a decrease of plaques in patients treated with CAD‐106 for 78 weeks.^[^ ^10^ ^]^ In a separate phase II/III clinical trial, the efficacy of CAD‐106 was compared with a small‐molecule inhibitor of beta‐secretase‐1 (BACE‐1). Although no cases of meningoencephalitis with CAD‐106 occurred,^[^ ^7^ ^]^ the trial was ended prematurely because of adverse effects in the control (BACE‐1) group and further development of CAD‐106 was discontinued.^[^ ^11^, ^12^ ^]^

ACI‐24 (AC Immune, Roche and Genentech) is designed to avoid eliciting a Th‐1 response to Aβ. ACI‐24 is made from Aβ_1–15_ anchored to a liposome by tetra‐palmitoylated lysine followed by a polyethylene glycol spacer on each end which allows anchoring to liposomes (Figure 1b).^[^ ^13^ ^]^ This liposome peptide delivery system, termed a SupraAntigen platform, was developed by AC Immune.^[^ ^14^ ^]^ CD spectroscopy established that the anchored peptide adopts an ordered β‐sheet conformation and thus indicates that the antigen is conformationally defined.^[^ ^13^ ^]^ In 2016, ACI‐24 was the first Aβ peptide vaccine used against AD in Down syndrome.^[^ ^15^ ^]^ Although a phase II clinical trial was scheduled to be completed in 2024, the trial was withdrawn in 2021 to continue optimizing vaccine formulation and improve study design.^[^ ^15^, ^16^ ^]^

ABvac40 (Axon Neuroscience SE) is designed to target the *C*‐terminus of Aβ_1–40_. Aβ_1–40_ is the predominant alloform of Aβ, and elevated levels of Aβ_40_ are correlated with AD severity.^[^ ^17^ ^]^ This peptide vaccine contains Aβ_33–40_ conjugated with the carrier protein keyhole limpet hemocyanin (KLH). In a phase I clinical trial, 11 out of 12 immunized patients produced antibodies against Aβ_1–40_. None of the patients developed amyloid‐related imaging abnormalities (ARIA), involving edema (ARIA‐E), microhemorrhage (ARIA‐H), or other signs of brain pathology.^[^ ^17^ ^]^ A phase II clinical trial is currently ongoing and is expected to be completed by the end of 2022.^[^ ^18^ ^]^

UB‐311 (Vaxxinity) targets the *N*‐terminus of Aβ and is made with the UBITh platform technology developed by United Biomedical.^[^ ^19^, ^20^ ^]^ UB‐311 contains a mixture of two different Aβ_1–14_ peptide antigens, both designed to induce a helper T‐cell response.^[^ ^21^ ^]^ In one, Aβ_1–14_ is linked to measles virus fusion protein (MVF_288–302_); in the other, Aβ_1–14_ is linked to the surface antigen from a hepatitis B virus (HBsAg_19–33_). These peptide conjugates are mixed with polyanionic CpG oligodeoxynucleotide to form micron‐sized immunostimulatory complexes and then with the Adju‐Phos adjuvant to create a Th‐2 biased peptide vaccine.^[^ ^21^ ^]^ In phase I, IIA, and IIB clinical trials, patients treated with UB‐311 tolerated the peptide vaccine and did not show any signs of ARIA‐E.^[^ ^22^ ^]^ Although phase II clinical trials were terminated in 2019 due to a treatment design error, there are plans for an additional phase IIB clinical trial.^[^ ^23^, ^24^ ^]^

## 3 TAU PEPTIDE VACCINES

**FIGURE 2:**
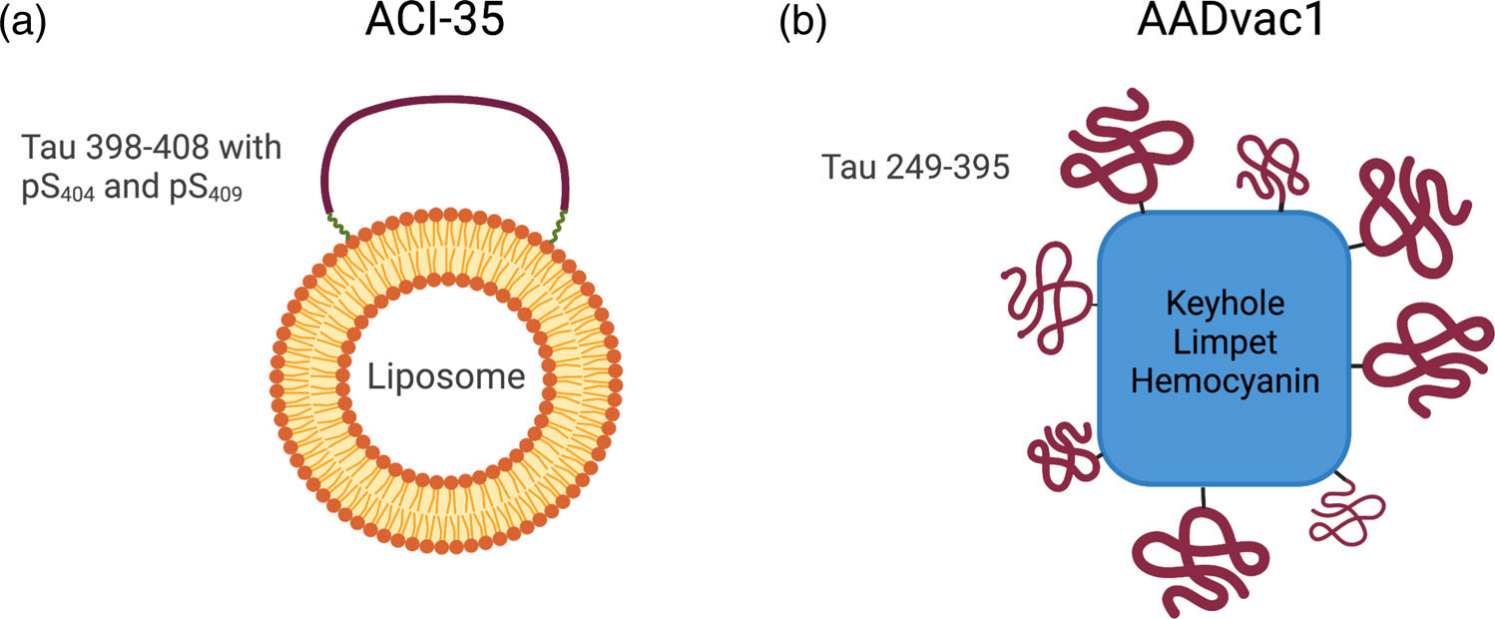
Two tau peptide vaccines that have advanced to phase II clinical trials. (a) ACI‐35 and (b) AADvac1

Tau peptide vaccines were developed in response to the discouraging outcomes of earlier Aβ peptide vaccines and immunotherapies. The AN‐1792 peptide vaccine showed modest clearance of tau and immunotherapies that target Aβ failed to significantly alter cerebrospinal fluid (CSF) levels of tau.^[^ ^4^ ^]^ Preclinical studies have focused on developing tau peptide vaccines against pathological forms of tau. However, identifying epitopes that produce therapeutic antibodies specific to pathological forms of tau remains challenging. Figure 2 summarizes the two tau peptide vaccine candidates tested in clinical trials (ACI‐35 and AADvac1) and illustrates the regions of tau used as antigens and the means of antigen presentation.

One of the first tau peptide vaccines was created by Asuni *et al*. and was tested in an AD tau mouse model.^[^ ^25^ ^]^ The vaccine consists of tau_393–408_ with phosphorylated serine at positions 396 and 404 in Adju‐Phos adjuvant. P301 transgenic AD mice immunized with the vaccine showed decreased aggregated tau in the brain, increased anti‐tau antibody titers, and slowed cognitive decline. The same peptide epitope was then used to create the ACI‐35 vaccine, which is one of only two tau peptide vaccines to enter clinical trials.^[^ ^26^ ^]^ This tau peptide vaccine was created with the same liposome‐based vaccine technology used to make ACI‐24.^[^ ^27^ ^]^

In ACI‐35 (AC Immune and Janssen), AC Immune's SupraAntigen platform takes the phosphorylated tau_398–408_ peptide and flanks the peptide by pairs of lipid‐bearing lysine residues thus anchoring the peptide to the liposome (Figure 2a). CD spectroscopy established that the anchored peptide adopts an ordered β‐sheet conformation and thus indicates that the antigen is conformationally defined.^[^ ^28^ ^]^ Phase IB clinical trials in 2013 tested AC1–35 for safety, tolerability, and therapeutic efficacy. The vaccine formulation elicited only a weak immune response, even with the administration of booster shots.^[^ ^28^, ^29^ ^]^ An improved formulation of the vaccine was subsequently designed, ACI‐35.030, with a second adjuvant and helper T‐cell epitopes. The ACI‐35.030 vaccine provided increased immune response in rhesus monkeys. Phase IB/IIA clinical trials began in August 2019, and results thus show high titers and antibodies specific for phosphorylated tau and aggregated tau.^[^ ^30^, ^31^ ^]^

AADvac1 (Axon Neuroscience) was inspired by epitopes recognized by the tau antibody, DC8E8.^[^ ^32^, ^33^, ^34^ ^]^ Epitope mapping studies, competition assays, and X‐ray crystallography revealed that DC8E8 binds to the amino acid sequence HXPGGG, which is found in the microtubule binding region of the 3R and 4R tau isoforms.^[^ ^4^ ^]^ In AADvac1, tau_294–395_ of the microtubule binding region is conjugated to KLH and formulated with Adju‐Phos adjuvant.^[^ ^35^ ^]^ Tau transgenic rats that were immunized with AADvac1 exhibited a Th‐2 immune response and high levels of anti‐tau antibodies. In 2013 phase I clinical trial, patients developed titers against AADvac1 and had no signs of brain inflammation.^[^ ^36^, ^37^ ^]^ Phase II clinical trials ended in 2019 and assessed for long‐term safety, tolerability, and efficacy in patients.^[^ ^38^, ^39^ ^]^ AADvac1 was safe and well‐tolerated but did not demonstrate improvement in cognitive impairment.^[^ ^40^ ^]^

## 4 β‐AMYLOID IMMUNOTHERAPIES

**FIGURE 3:**
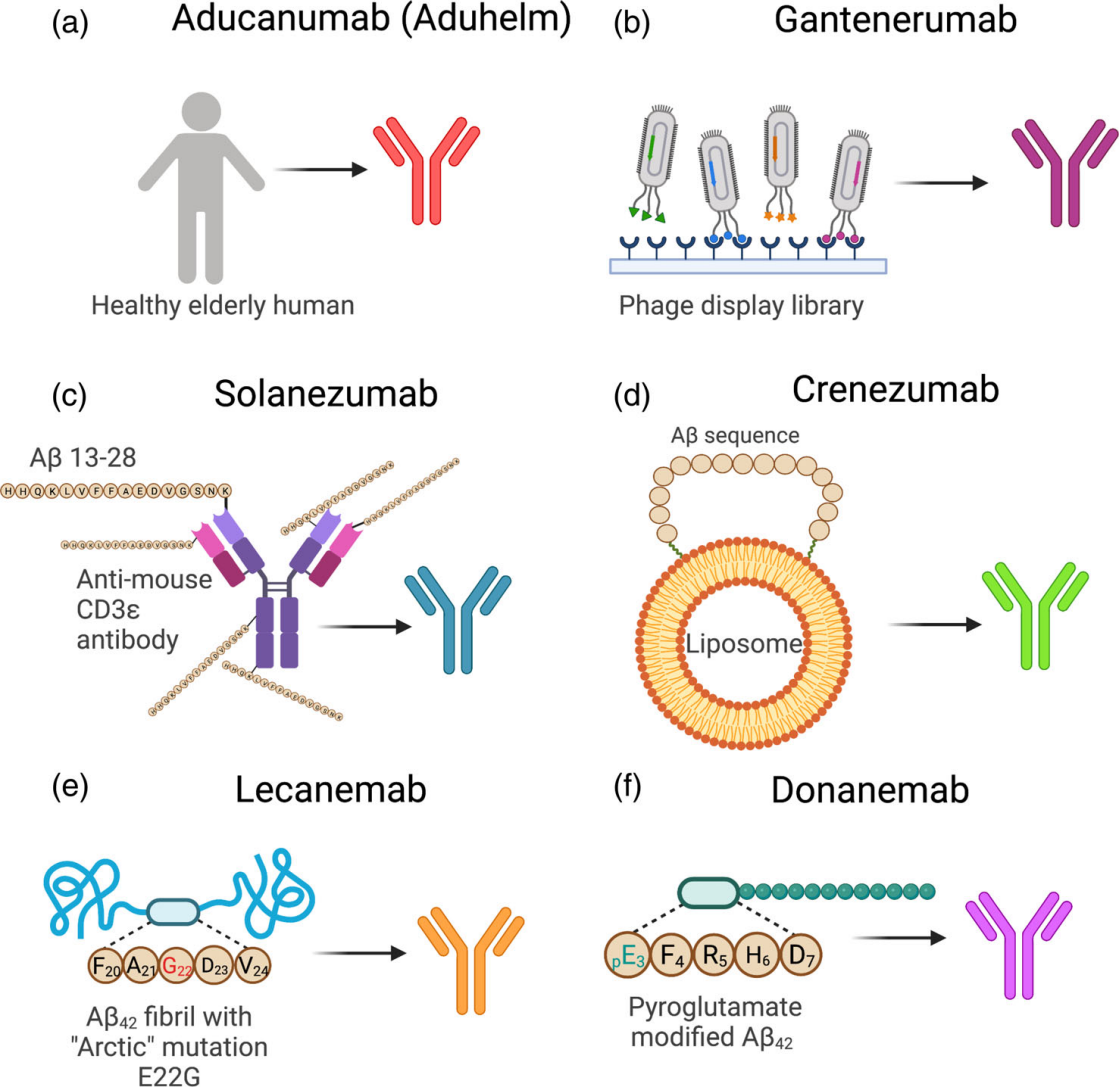
Derivation of six Aβ immunotherapies. (a) Aducanumab, (b) Gantenerumab, (c) Solanezumab, (d) Crenezumab, (e) Lecanemab, and (f) Donanemab

Two decades of effort have led to the controversial FDA approval of the Aβ monoclonal antibody Aducanumab (Aduhelm), which has subsequentially sparked the revival of three additional monoclonal antibodies against Aβ (Gantenerumab, Solanezumab, and Crenezumab) that were previously terminated in the drug pipeline. Two additional anti‐Aβ monoclonal antibodies, Donanemab and Lecanemab, are now in phase III clinical trials. Figure 3 illustrates the antigen that was used to generate each of these monoclonal antibodies. Additional details are summarized in Table S2.

Aducanumab (Aduhelm: Biogen, Eisai, Neurimmune) was derived from B‐cells of healthy elderly donors that display antibodies that recognize aggregated Aβ (Figure 3a), and its discovery was driven by the rationale that the cognitively normal patients had antibodies that could benefit AD patients.^[^ ^41^ ^]^ The FDA approved Aducanumab in June 2021 as the first drug in its class to treat AD.^[^ ^42^ ^]^ The approval was controversial, because Aducanumab had only marginal effects on cognitive impairment despite clearing plaques, and because it displayed side effects in a large percentage of patients (ARIA, confusion, dizziness, nausea, and headache).^[^ ^43^, ^44^, ^45^, ^46^, ^47^ ^]^ For these reasons, Aducanumab has not been widely adopted as a treatment for AD and has been rejected by many medical providers,^[^ ^48^ ^]^ insurance companies,^[^ ^49^, ^50^ ^]^ and international drug and regulatory agencies.^[^ ^51^ ^]^ Biogen plans to submit a final protocol to perform phase IV clinical trials for FDA review. The approval of Aducanumab has revived previously terminated monoclonal antibodies and reinvigorated efforts to develop immunotherapies against AD.^[^ ^52^ ^]^

Gantenerumab (Roche and Chugai Pharmaceutical) was generated to target and sequester Aβ plaques by stimulating microglia to clear Aβ through phagocytosis. Gantenerumab was created from a synthetic human antibody phage display library and *in vitro* maturation on Aβ fibrils (Figure 3b).^[^ ^53^ ^]^ In October 2021, the FDA granted accelerated development and review in a fashion similar to Aducanumab.^[^ ^54^ ^]^ Two active phase III clinical trials^[^ ^55^, ^56^ ^]^ and one phase II^[^ ^57^ ^]^ clinical trial are currently underway to assess the efficacy of Gantenerumab, with the phase II clinical trial and one of the phase III clinical trials^[^ ^56^ ^]^ assessing in prodromal or mild AD patients. Two open‐label phase III clinical trials are actively recruiting patients and are expected to end in 2024^[^ ^58^ ^]^ and 2026.^[^ ^59^ ^]^ Washington University in St. Louis has initiated a collaboration with Roche to test Gantenerumab on patients with mutations that cause AD at as young as 30 years old, making it the first monoclonal antibody to be tested in such a young demographic of patients.^[^ ^60^ ^]^

Solanezumab (Eli Lilly) is a humanized IgG1 monoclonal antibody against Aβ that was derived from a murine precursor raised against the central region of Aβ. The murine antibody was generated by immunizing mice with Aβ_13–28_ conjugated to an antimouse CD3*ε* antibody using the heterobifunctional crosslinker MBS and formulated in Freund's complete adjuvant (Figure 3c).^[^ ^61^ ^]^ Although four phase III clinical trials were completed in 2012^[^ ^62^, ^63^ ^]^ and terminated in 2017,^[^ ^64^, ^65^ ^]^ one phase III clinical trial assessing the efficacy in slowing plaque production and cognitive impairment will be completed in 2023.^[^ ^66^ ^]^ Phase II/III clinical studies comparing the efficacy of Gantenerumab and Solanezumab showed that these monoclonal antibodies failed to reverse cognitive impairment.^[^ ^67^ ^]^ Despite these previous results, another trial is recruiting patients and is expected to be completed in 2022.^[^ ^68^ ^]^

Crenezumab (Genentech and Roche) is a humanized IgG4 antibody selected to target Aβ while preventing over‐activation of microglia‐mediated brain inflammation.^[^ ^69^ ^]^ Crenezumab was generated by immunizing AD transgenic mice with an Aβ peptide displayed on AC Immune's SupraAntigen platform (Figure 3d).^[^ ^13^, ^28^, ^69^ ^]^ Three phase III clinical trials were terminated in 2019 because interim analysis suggested that the treatment was unlikely to meet clinical endpoints.^[^ ^66^, ^67^, ^68^, ^70^ ^]^ Current efforts in accessing the therapeutic potential of Crenezumab have shifted to patients with familial AD. A phase II clinical trial evaluating the safety and efficacy of Crenezumab in familial AD patients began in 2013 and is expected to be completed in 2022.^[^ ^71^ ^]^ A subsequent phase II clinical trial using PET imaging to access for tau burden in treated patients was initiated in 2019. This study is continuing to recruit patients and is planned to be completed by March 2022.^[^ ^72^ ^]^

Lecanemab (Biogen, Eisai, and BioArctic) is an IgG1 monoclonal antibody against Aβ that was derived from a murine antibody generated against the “Arctic” mutation E22G of Aβ, which leads to high levels of fibrils without plaque deposition.^[^ ^73^, ^74^, ^75^, ^76^ ^]^ The murine antibody was created by immunizing mice with E22G Aβ fibrils in Freund's complete adjuvant (Figure 3e).^[^ ^76^ ^]^ In a phase IIB clinical trial, which is expected to be completed by 2025,^[^ ^77^ ^]^ treatment reduced 93% of plaques and slowed cognitive decline by 27%–56%, as assessed by multiple statistical models.^[^ ^78^ ^]^ A phase III clinical trial is ongoing accessing safety and efficacy in patients with early AD and is expected to end in 2024.^[^ ^79^ ^]^ Two weeks after the FDA approval of Aducanumab, the FDA granted accelerated approval of Lecanemab in early AD.^[^ ^80^ ^]^ The FDA subsequently granted “Fast Track Designation” to further expedite development.^[^ ^81^ ^]^ Phase II/III clinical trials are currently assessing patients with familial AD receiving either the Aβ antibody Lecanemab or the tau antibody E2814 and are expected to end October 2027.^[^ ^82^, ^83^ ^]^

Donanemab (Eli Lilly) is the humanized IgG1 monoclonal antibody of a murine antibody that was developed to specifically target existing Aβ plaques, rather than prevent plaque development. In contrast to previous Aβ monoclonal antibodies, the mice were immunized with a pyroglutamate form of Aβ found in aggregated Aβ (Aβ_p3–42_ peptide, Figure 3f).^[^ ^84^ ^]^ Donanemab is currently in a phase III clinical trial assessing AD patients with prodromal to mild AD^[^ ^85^ ^]^ and has been granted accelerated approval, in a fashion similar to Aducanumab and Lecanemab.^[^ ^86^ ^]^ Two additional phase III clinical trials are recruiting patients, with one trial treating patients at risk of cognitive decline with AD^[^ ^87^ ^]^ and the other comparing plaque clearance in patients treated either with Donanemab or Aducanumab.^[^ ^88^ ^]^

## 5 TAU IMMUNOTHERAPIES

**FIGURE 4:**
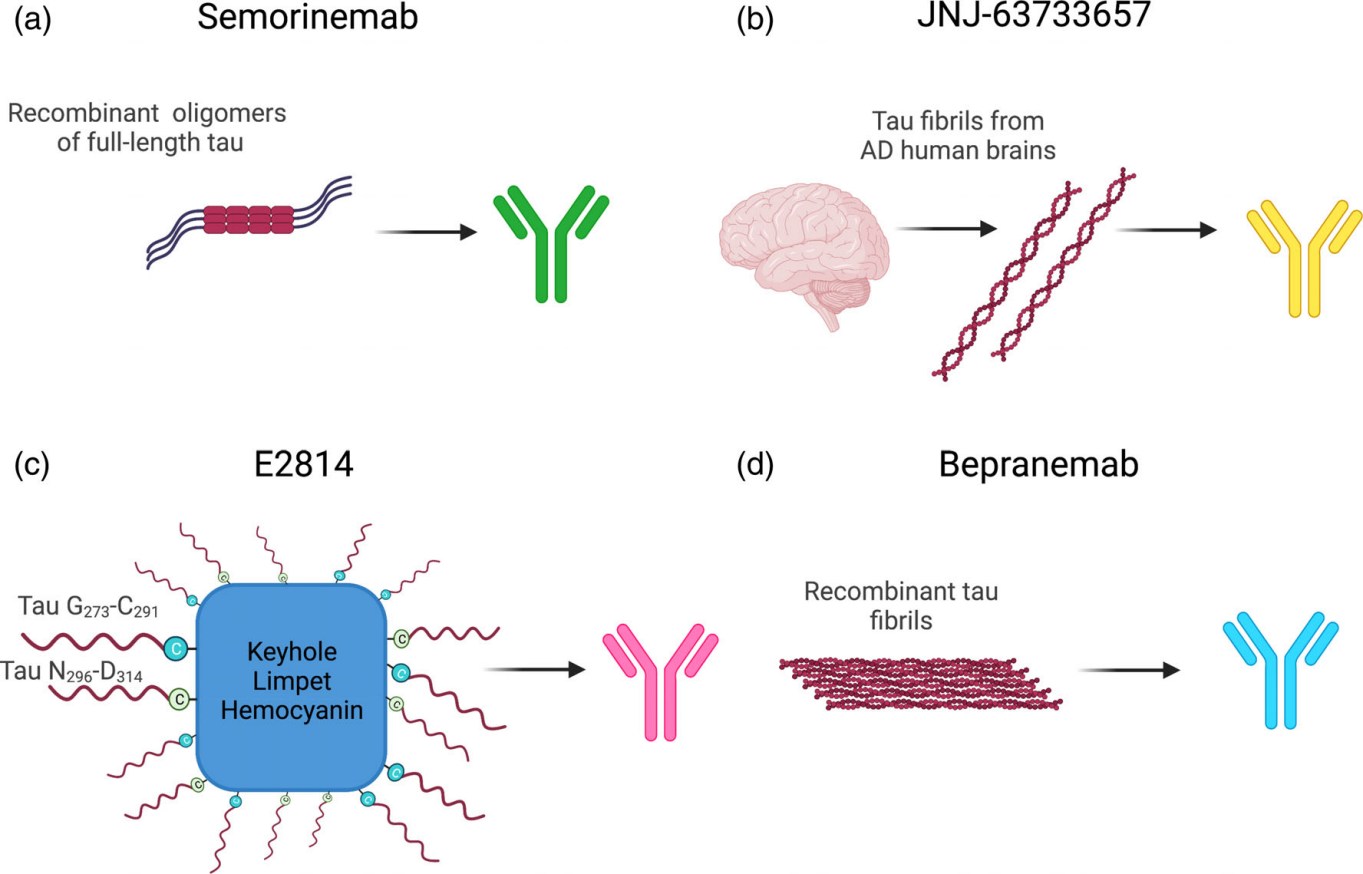
Derivation of four tau immunotherapies. (a) Semorinemab, (b) JNJ‐63733657, (c) E2814, and (d) Bepranemab

Monoclonal antibody immunotherapies against tau are starting to show promise in treating AD, with four notable examples in or about to be in phase II clinical trials (Semorinemab, JNJ‐63733657, E2814, and Bepranemab). Figure 4 illustrates the antigen that was used to generate each of these monoclonal antibodies. Additional details are summarized in Table S3.

Semorinemab (AC Immune SA, Genentech, and Roche) is a humanized IgG4 antibody against tau that was designed to reduce microglia‐mediated brain inflammation though mutation of the Fc region. Its murine precursor was derived from oligomers generated from recombinant full‐length human tau isoform 2N4R (Figure 4a).^[^ ^89^, ^90^ ^]^ In a 2017 phase II clinical trial, 0.3% of Semorinemab was found to enter the CSF, and the concentration of *N*‐terminal tau in the CSF increased, suggesting that the antibody was targeting tau.^[^ ^91^, ^92^ ^]^ Nevertheless, Semorinemab did not decrease markers of neurodegeneration and inflammation.^[^ ^93^ ^]^ The launch of a phase III clinical trial is pending on results from a separate 2019 phase II clinical study that will be completed in 2023.^[^ ^94^ ^]^

JNJ‐63733657 (Janssen) is a humanized IgG1 monoclonal antibody that was selected for targeting the microtubule‐binding region of tau and disrupting cell‐to‐cell propagation of aggregated tau.^[^ ^74^, ^95^ ^]^ Its murine precursor was derived by immunizing mice with tau paired‐helical filaments isolated from AD human brain (Figure 4b).^[^ ^81^, ^96^ ^]^ JNJ‐63733657 was tested in two phase I clinical trials. The 2017 phase I clinical trial reported the monoclonal antibody to be safe and well tolerated. The 2019 phase I clinical trial reported ~0.2% of JNJ‐63733657 entering into the CSF and a dose‐dependent reduction of pS_217_ tau.^[^ ^93^ ^]^ A phase II clinical trial began in January 2021 and is expected to run until 2025.^[^ ^97^ ^]^

E2814 (Eisai) is a humanized IgG1 monoclonal antibody that was selected for its affinity to the microtubule binding region of tau.^[^ ^98^ ^]^ Its murine precursor was generated by immunizing transgenic mice with two 19‐mer peptide fragments from the 4R tau isoform that is essential for tau seeding and aggregation. The fragments (G_273_–C_291_ and N_296_–D_314_ with an *N*‐terminal cysteine) contain the R2 and R3 repeats and are designed to present key epitopes and to coassemble and thus minimize further aggregation. The fragments were conjugated to KLH and administered with Freund's complete adjuvant (Figure 4c).^[^ ^98^ ^]^ A phase I clinical trial began in December 2019 and is expected to be completed in November 2022.^[^ ^96^ ^]^ A phase I/II clinical trial began in June 2021 and is currently recruiting patients.^[^ ^99^ ^]^

Bepranemab (UCB Biopharma SRL) is a humanized IgG4 monoclonal antibody selected for its efficacy in blocking human tau seeds *in vitro* and was generated by immunizing Sprague–Dawley rats with fibrils of recombinant tau (Figure 4d).^[^ ^26^, ^100^ ^]^ Bepranemab was originally tested in phase I clinical trials for safety, tolerability, and efficacy against progressive supranuclear palsy.^[^ ^101^, ^102^ ^]^ It is now being tested in a phase II clinical trial for AD, which is expected to be completed in 2025.^[^ ^103^ ^]^

## 6 CONCLUSION AND PERSPECTIVE

More than two decades of effort to develop peptide vaccines and monoclonal antibody immunotherapies that target Aβ and tau have revealed that there is much left to be explored to create safe and effective peptide vaccines and immunotherapies against AD. Although peptide vaccines may hold the ultimate promise of being widely administered to prevent AD, their development has proven challenging because of adverse reactions such as ARIA. Immunotherapies are currently showing the most immediate promise in spite of being costly and requiring repeated intravenous administration. Aducanumab currently costs $56,000 per year,^[^ ^49^, ^50^ ^]^ requires high dosing (10 mg/kg) every 4 weeks,^[^ ^104^ ^]^ has marginal efficacy, and risks inducing ARIA. It is now thought that beginning treatment early is important for slowing or preventing the decline associated with AD.^[^ ^105^ ^]^ For this reason, some of the current clinical trials are focusing on patients with mild or prodromal AD. Other clinical trials have focused on younger patients who are at risk of familial AD^[^ ^60^ ^]^ or who have Down syndrome.^[^ ^15^ ^]^

A major limitation of the immunotherapies that have been developed thus far is that antibodies do not easily cross the blood–brain barrier and only small amounts (<1%) of the antibodies administered enter the brain. New technology for antibody delivery to the brain may allow the development of more efficacious immunotherapies. Antibodies that bind to the transferrin receptor can cross the blood–brain barrier by transcytosis, binding to the transferrin receptor and then being transported across the epithelial cells lining blood vessels in the brain.^[^ ^106^ ^]^ Roche has thus created a derivative of Gantenerumab (RO7126209) that enters the CSF with eightfold greater efficacy by conjugating Gantenerumab to a Fab fragment that binds to the transferrin receptor. In phase I clinical trials, this bioconjugate showed no signs of inducing ARIA.^[^ ^107^ ^]^ Phase IB/IIA clinical trials are ongoing and are expected to be completed by 2024.^[^ ^108^ ^]^ Other approaches to targeting the transferrin receptor for improved transport of antibodies have also been developed.^[^ ^109^ ^]^

Additional improvements in immunotherapies may emerge. Apolipoprotein E (APOE) is another promising target for monoclonal antibody immunotherapies in AD that has begun to be explored in preclinical trials.^[^ ^110^, ^111^, ^112^ ^]^ Combination therapies involving multiple immunotherapies or immunotherapies in combination with other drugs are also being explored.^[^ ^82^, ^113^ ^]^

Even if monoclonal antibody immunotherapies become safer and more efficacious, it is unlikely that they will be widely administered at an early enough age to prevent AD, because they will likely require repeated intravenous administration starting in middle age. In this sense, immunotherapies are unlikely to ever be used widely, like drugs for high blood pressure or high cholesterol. Nevertheless, monoclonal antibody immunotherapies are likely to continue to be developed, we hope with increased efficacy. In the long run, we hope that safe and efficacious peptide vaccines can be created for prophylaxis in the broad population of middle‐aged adults.

Patients receiving Aβ peptide vaccines are typically immunized with the *N*‐terminus of Aβ because it is a B‐cell epitope. Although the middle region of Aβ, between residues 16 and 32, has been avoided out of concerns that it can induce a Th‐1 response, it deserves further study, particularly as the development of Th‐2 biased vaccine formulations continues to advance.^[^ ^3^ ^]^ This region of Aβ can present unique peptide epitopes in loop‐like conformations when folded into β‐hairpins, which are thought to make up the toxic amyloid oligomers associated with neurodegeneration.^[^ ^114^, ^115^, ^116^ ^]^ Peptide vaccines containing the middle region of Aβ in β‐hairpin conformations may thus lead to conformation‐specific antibodies with potential to bind and detoxify these amyloid oligomers. The epitopes presented by this region of Aβ should be distinct from those presented by the *N*‐terminal region of Aβ, which can also fold into a β‐hairpin‐like structure.^[^ ^117^, ^118^, ^119^, ^120^ ^]^

Much work also remains to be done in the development of tau vaccines, because there are a multitude of different hyperphosphorylated tau epitopes that need to be explored.^[^ ^121^ ^]^ Tau contains more than 80 potential sites of phosphorylation on serine, threonine, and tyrosine residues,^[^ ^122^ ^]^ which create a daunting challenge in selecting and exploring biologically relevant antigens.^[^ ^123^, ^124^, ^125^ ^]^ Exploration of these antigens may ultimately provide a better molecular understanding of AD, which can further guide peptide vaccine and monoclonal antibody immunotherapy development.

## CONFLICTS OF INTEREST

James S. Nowick serves as an Advisory Board member of Peptide Science, and was excluded from both the peer‐review process and all editorial decisions related to the publication of this article. We have no additional conflicts of interest.

## Supporting information


**TABLE S1** Summary of Aβ peptide vaccines.
**TABLE S2**. Summary of Aβ passive immunotherapies.
**TABLE S3**. Summary of tau passive immunotherapies

## Data Availability

Data sharing is not applicable to this article as no new data were created or analyzed in this study.
