# Supplementary material for: Current peptide vaccine and immunotherapy approaches against Alzheimer's disease
Source: Pept Sci (Hoboken). 2022 Jun 24;115(1):e24289. doi: 10.1002/pep2.24289 (PMC9916509; doi:10.1002/pep2.24289)
Supplement: Supplementary file 1 — TABLE S1 Summary of Aβ peptide vaccines. TABLE S2. Summary of Aβ passive immunotherapies. TABLE S3. Summary of tau passive immunotherapies [file PEP2-115-0-s001.pdf]

Supporting Information for

Current Peptide Vaccine and Immunotherapy Approaches Against Alzheimer's Disease.

Chelsea Marie T. Parrocha[b] and James S. Nowick[*a,b]

[a]Department of Chemistry, University of California Irvine, Irvine, CA 92697

[b]Department of Pharmaceutical Sciences, University of California Irvine, Irvine, CA 92697

Table S1. Summary of Aβ peptide vaccines.

Vaccine	Sponsor	Antigen	Adjuvant	Carrier Molecules and Crosslinker	Immune Response by adjuvant	Phase in Clinical Research
Amilomotide (CAD 106)	Novartis Pharmaceuticals	Aβ 1–6	Alum or MF59	Bacteriophage Qbeta (VLP Qβ phage)<br><br>Malamide	Th2	Phase II/III Discontinued
ACI-24 (Pal 1–15 acetate salt)	AC Immune, Roche, and Genentech	Aβ 1–15	MPLA	Liposomes with tetra-palmitoylated lysines	Th1/Th2	Phase II Withdrawn
ABvac40	Axon Neuroscience SE	$A\beta_{40}$ 33–40	Alum	Keyhole Limpet Hemocyanin	Th2	Phase II Active, not recruiting
UB-311	United Biomedical (Vaxxinity)	Aβ 1–14	Alum + CpG	Measles virus fusion protein (288–302)<br><br>Hepatitis B virus surface antigen (19–33)<br><br>UBITh platform technology	Th1/Th2	Phase II Terminated
Affiris AD02 (AffitopeAD02)	AFFiRiS and GlaxoSmithKline	Aβ 1–6	Alum	Keyhole Limpet Hemocyanin<br><br>Malmaide	Th2	Phase II Recruiting
Vanutide cridificar (ACC-001, PF-05236806)	Jansen, Pfizer	Aβ 1–7	QS-21	Diphtheria Toxin, Thioether (CRM197)	Th1/Th2	Phase II 2 Terminated and 7 Completed between 2013–2014
V950	Merk	Aβ 1–15	Quil A	ISCOMATRIX	Th1/Th2	Phase I Completed
Mimovax<br><br>(Affitope AD03)	AFFiRiS GlaxoSmithKline	Pyroglutamate modified N-terminal Aβ	Alum	Keyhole Limpet Hemocyanin	(Assumpti on) Th2	Phase I Completed
Lu AF20513	Lundbeck/Otsuka	Aβ 1–12	Alum	Th epitopes (P2P2 & P30 from Tetanus)	Th2	Phase I Terminated 2019
ALZ-101	Alzinova	Proprietary formulation of cross-linked Aβ	N/A	N/A	N/A	Phase I Recruiting

Table S2. Summary of Aβ passive immunotherapies.

Antibody	Sponsor	Epitope Recognition	Derivation	Phase in Clinical Research
Aducanumab (BIIB037, Aduhelm)	Biogen, Eisai, and Neurimmune	Aβ 3–7	B cell library from health elderly patients	Phase III Active, not recruiting
Gantenerumab (RO4909832, RG1450)	Chugai Pharmaceutical and Roche	Aβ 2–11 and 18-27	Human antibody phage display library	Phase III Recruiting
Solanezumab (LY2062430)	Eli Lilly	Aβ 16–26	Aβ 13–28	Phase III Active, not recruiting
Crenezumab (MABT5102A, RG7412)	Genentech and Roche	Aβ 13–24	Liposome anchored peptides	Phase II Recruiting
Lecanemab (BAN2401, mAb158)	Biogen, Eisai, and BioArctic	Aβ 1–16	Fibrils of E22G mutant of Aβ	Phase III Recruiting
Donanemab (N3pG-Aβ, LY3002813, mE8)	Eli Lilly	Aβ p3–7	Aβ pE3-42 peptide	Phase III Recruiting
Bapineuzumab (AAB-001, 3D6)	Janssen, Pfizer	Aβ 1–5	Aβ 1–5 conjugated to an antibody	Phase III Terminated
MEDI1814	AstraZeneca, Eli Lilly	$A\beta_{42}$ 29-42	Human antibody phage display library	Phase I Completed
Ponezumab (PF-04360365)	Janssen, Pfizer	Aβ 30-40	$A\beta_{40}$	Phase II Completed
RO7126209 (RG6102, Brain shuttle gantenerumab)	Roche	N/A	Gantenerumab conjugated to a Fc region that binds to the transferrin receptor	Phase I/II Recruiting
SAR228810 (SAR255952, 13C3)	Sanofi	4–20	Synthetic oligomers	Phase I Complete
AAB-003 (PF-05236812)	Janssen, Pfizer	1–8 and 1–28	Bapineuzumab with modified Fc region	Phase 1 Completed
GSK933776	GlaxoSmithKline	N-terminus of Aβ	Modified Fc region of an IgG1 antibody	Phase II Completed
LY2599666	Eli Lilly	Mid-region of Aβ	anti-Aβ Antigen-binding fragment linked to modified Fc region with polyethylene glycol	Phase I Terminated
LY3372993	Eli Lilly	N/A	N/A	Phase I Recruiting

Table S3. Summary of tau passive immunotherapies.

Antibody	Sponsor	Epitope Recognition	Derivation	Phase in Clinical Research
Semorinemab (RO7105705, MTAU9937A, RG6100)	AC Immune SA, Genentech, and Roche	Tau 2–24	Recombinant oligomers	Phase II Active, not recruiting
JNJ-63733657 (B296, PT3)	Janssen	Tau 204–225 (pT212–pT217)	Tau fibrils from patients	Phase II Recruiting
E2814	Eisai	HVPGG sequence and 299–303, and 362–366	Tau fragments G273–C291 and N296–D314 with an N-terminal cysteine	Phase II/III Recruiting
Bepranemab (UCB0107, Antibody D, D IgG4)	Roche, UCB S.A.	Tau 235–246	Tau recombinant fibrils	Phase II Recruiting
Tilavonemab (ABBV-8E12, C2N 8E12, HJ9.3)	AbbVie, C2N Diagnostics	Tau 25–30 and 22–34	Recombinant full length tau	Phase II Terminated
Gosuranemab (BIIB092, BMS-986168, IPN007/IPN002)	iPerian, Biogen, Bristol-Myers Squibb	Tau 15–24 and 8–19	*in vitro* aggregated full length tau	Phase II Terminated
Zagotenemab (LY3303560, MC-1 IgG1)	Eli Lilly & Co.	Tau 7–9 and 312–322	Immunopurified paired helical filaments	Phase II Completed
Lu AF87908	H. Lundbeck A/S	pS396	Tau 386–408 with pS390 and 404	Phase I Recruiting
BIIB076 (NI-105, 6C5 huIgG1/l)	Biogen, Eisai, and Neurimmune	Mid-region	Proprietary platform	Phase I Completed
RG7345 (RO6926496)	Roche	416–430, pS422	416–430, pS422 peptide	Phase I Completed
